# Chemokine-Induced Macrophage Polarization in Inflammatory Conditions

**DOI:** 10.3389/fimmu.2018.01930

**Published:** 2018-09-07

**Authors:** Pieter Ruytinx, Paul Proost, Jo Van Damme, Sofie Struyf

**Affiliations:** Laboratory of Molecular Immunology, Department of Microbiology and Immunology, REGA Institute KU Leuven, Leuven, Belgium

**Keywords:** macrophage polarization, chemokines, tumor-associated macrophage, leukocyte migration, inflammation and cancer

## Abstract

Macrophages represent a heterogeneous cell population and are known to display a remarkable plasticity. In response to distinct micro-environmental stimuli, e.g., tumor stroma vs. infected tissue, they polarize into different cell subtypes. Originally, two subpopulations were defined: classically activated macrophages or M1, and alternatively activated macrophages or M2. Nowadays, the M1/M2 classification is considered as an oversimplified approach that does not adequately cover the total spectrum of macrophage phenotypes observed *in vivo*. Especially in pathological circumstances, macrophages behave as plastic cells modifying their expression and transcription profile along a continuous spectrum with M1 and M2 phenotypes as extremes. Here, we focus on the effect of chemokines on macrophage differentiation and polarization in physiological and pathological conditions. In particular, we discuss chemokine-induced macrophage polarization in inflammatory diseases, including obesity, cancer, and atherosclerosis.

## Introduction

Monocytes arise in the bone marrow from hematopoietic stem cells (HSCs) and develop through a series of sequential differentiation stages. Common myeloid progenitor cells develop into granulocyte/macrophage colony forming units (GM-CFU), which in turn can commit to the macrophage colony-forming unit (M-CFU) or the granulocyte colony-forming unit (G-CFU). The M-CFU differentiates sequentially into monoblasts and promonocytes, which leave the bone marrow and enter the bloodstream, where they differentiate into mature monocytes (1). Mature monocytes represent about 10% of the leukocyte population in human peripheral blood and can circulate in the blood stream for up to 1–2 days before they undergo apoptosis. Alternatively, monocytes can migrate into the tissues and differentiate into specific macrophages (2). The major driver for the homeostatic control of monocyte/macrophage development is macrophage colony-stimulating factor (M-CSF), present in the blood circulation and produced by stromal cells in tissues (3–5). In inflammatory conditions, also other cytokines such as granulocyte-macrophage colony-stimulating factor (GM-CSF) and the chemokine CXCL4 influence the differentiation and/or survival of mononuclear phagocytes (6–8).

In contrast to the classical model of macrophage development, where macrophages differentiate from circulating monocytes as described above, recent studies provided evidence that tissue-resident macrophages arise from yolk sac or fetal liver-derived progenitors (9). These tissue resident macrophages appear to have stem cell-like capacities as they persist independently of monocytes by self-renewal *in situ* (10). One of the major hallmarks of macrophages is their heterogeneity, which is reflected by their specialized function in a particular microenvironment. According to their tissue location, macrophages can take different names including microglia [central nervous system (CNS)], Kupffer cells (liver), alveolar macrophages (lung), osteoclasts (bone), histiocytes (spleen and connective tissue), Langerhans cells (skin), and tissue macrophages in the gut (11). Resident macrophages promote tissue homeostasis, whereas monocyte-derived macrophages primarily assist in host-defense. Moreover, macrophages recruited during and after embryogenesis co-exist in different organs (10, 12).

Besides their heterogeneity, macrophages are known to display remarkable plasticity. In response to different micro-environmental stimuli, a fully differentiated macrophage can adopt a polarized phenotype with specific functional characteristics. Traditionally, macrophages are subdivided into two subpopulations: the classically activated or M1 macrophages and the alternatively activated or M2 macrophages (13). M1 macrophages can be induced by the Th1 cytokines tumor necrosis factor (TNF)-α, interferon (IFN)-γ and bacterial components such as lipopolysaccharide (LPS). Activated M1 macrophages phagocytose and destroy microbes, eliminate tumor cells and present antigens to T cells to evoke an adaptive immune response. As such, they play an important role in protection against pathogens. The pro-inflammatory phenotype is characterized by the increased production of reactive nitrogen intermediates (RNI) and reactive oxygen species (ROS), which is essential for bacterial killing (14). In response to inflammatory mediators, M1 macrophages express the inducible nitric oxide synthase (iNOS), which uses L-arginine as a substrate to produce nitric oxide (NO) (15). Furthermore, classically activated macrophages release high levels of pro-inflammatory cytokines such as TNF-α, interleukin-6 (IL-6) and IL-1β to deal with infections and thereby promote Th1 responses (16).

M2 activation occurs in response to stimulation with IL-4, IL-10, and IL-13. These macrophages display high surface levels of scavenger, mannose and galactose type receptors involved in debris clearance. Furthermore, they show a more immunosuppressive phenotype characterized by decreased antigen presentation to T cells and production of cytokines that stimulate a Th2 response. In contrast to M1 macrophages, M2 macrophages constitutively express the enzyme Arginase 1 (ARG1), which hydrolyzes L-arginine to L-ornithine (13). L-ornithine is the main precursor for polyamines, essential for cell survival. Furthermore, L-ornithine can also be used as a building block to make proline and hydroxyproline, essential amino acids for the production of collagen, a crucial protein in tissue damage repair (17). As such, these macrophages are involved in long-term tissue repair, promote tumor growth and exert antiparasitic effects (18).

Nowadays the M1/M2 classification is considered as an oversimplified approach that does not fully cover the total spectrum of *in vivo* macrophage phenotypes. Especially, in pathological circumstances macrophages behave as plastic cells modifying in space and time their expression and transcription profile along a continuous spectrum, having M1 and M2 macrophage phenotypes as extremes (19, 20).

The interaction of chemokine receptors on circulating cells with their ligands enables the selective tissue-specific recruitment of subsets of circulating cells such as monocytes. Chemokines are a family of low molecular weight, secreted proteins with a prominent role in leukocyte activation and chemotaxis. Based on the NH_2_-terminal motif of two conserved cysteine residues, chemokines can be classified into 4 subfamilies: C, CC, CXC, and CX_3_C chemokines. Chemokines signal via G protein-coupled receptors (GPCRs), which are named XCR, CCR, CXCR, CX_3_CR according to the chemokine nomenclature (21). Additionally, chemokines can bind with high affinity to atypical chemokine receptors (ACKRs), a subgroup of seven-transmembrane receptors highly related to the classical GPCRs. Since these ACKRs lack or have a modified canonical DRYLAIV motif, activation of ACKRs does not lead to typical GPCR-mediated signaling and chemotactic functions (22).

## The effect of chemokines on macrophage differentiation and polarization in physiological and pathological conditions

### Neurological diseases

Microglia, the resident, long-living macrophages in the central nervous system (CNS), act as the major inflammatory cell type in the brain and similar to peripheral macrophages they respond to pathogens and injury (23). Under physiological conditions, microglia are in a “quiescent” state or have a non-activated phenotype (24). Butofsky et al. demonstrated that this “resting” cell' phenotype is different from M1 or M2 microglia and expresses genes associated with neuronal development (25). This particular phenotype was found to be important for synaptic growth, maintenance, and neuronal growth. Furthermore, the “quiescent” state enables the intimate connection between neurons and microglial cells, which is tightly controlled by the CX_3_CL1-CX_3_CR1 axis (26). CX_3_CL1/fractalkine is the only member of the CX_3_C chemokine subfamily and differs from most other chemokines, as it can exist as a membrane-associated molecule with the chemokine motif being attached to a long mucin stalk. Alternatively, CX_3_CL1 is secreted as a soluble variant (27). CX_3_CL1 is expressed on healthy neurons, whereas the transmembrane protein receptor CX_3_CR1 is present on microglia (23, 28, 29).

**Figure 1:**
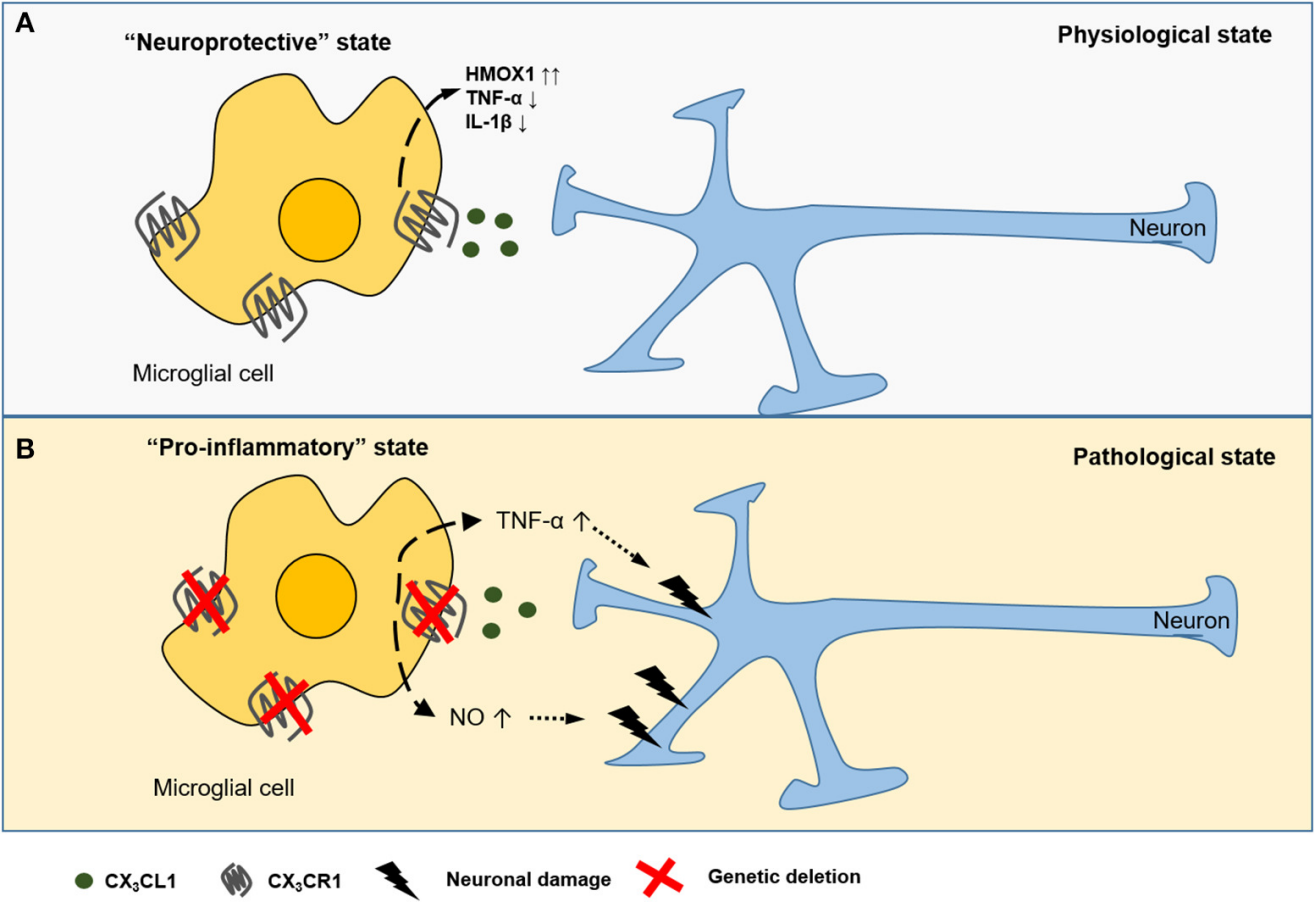
CX_3_CL1-CX_3_CR1 interaction between neurons and microglial cells in the CNS. CX_3_CL1 is released from the neurons and interacts with the CX_3_CR1 receptor expressed on CNS microglia. CX_3_CL1 signaling induces (dashed arrow) a neuroprotective state **(A)**, characterized by the suppressed release of pro-inflammatory cytokines (TNF-α, IL-1β) and upregulation of heme oxygenese 1 (HMOX1). In several murine models of neurodegenerative diseases, genetic deficiency of CX_3_CR1 is associated with potentially detrimental secretion of pro-inflammatory cytokines and reactive nitrogen species (NO) causing (dotted arrow) neurotoxicity **(B)**.

The CX_3_CL1-CX_3_CR1 axis is an important neuroimmune interaction in the CNS and has been implicated in many neurophysiological and neuropathological conditions (Figure 1). For instance, in animal models of Parkinson's disease and amyotrophic lateral sclerosis (ALS), loss of CX_3_CR1 increased neuronal cell death (30). Using a murine model of diabetic retinopathy, Cardona et al. showed that in the absence of CX_3_CR1 the microglial response is dysregulated and associated with increased IL-1β cytokine release (Figure 1B) (31). Additionally, Mattison et al. found that CX_3_CL1 suppressed the release of pro-inflammatory and neurotoxic factors such as TNF-α and NO in activated microglia during neuroinflammation (Figure 1B) (32). Controlling neuroinflammation via CX_3_CR1 signaling was particularly beneficial in the pathogenesis of Alzheimer's disease (33). Furthermore, CX_3_CL1 promotes microglial phagocytosis of neuronal debris and increases the expression of heme oxygenase 1 (HMOX1), resulting in an anti-oxidant effect, which indirectly promotes neuronal survival (34). Conversely, some studies showed a neurotoxic role for CX_3_CL1 in CX_3_CR1^−/−^ mice models for Alzheimer's disease (35) and stroke (36). Fuhrmann et al. also reported that neuronal loss neuronal loss in a model of Alzheimer's disease was prevented in CX_3_CR1 knock out mice (37).

The atypical chemokine receptor CCRL2 was identified as an important regulator of microglial activation and polarization in experimental autoimmune encephalomyelitis (EAE) (38). Similar to ACKRs, CCRL2 lacks conventional GPCR signaling and chemotactic activity (39). More specifically, it was found that during the chronic disease phase microglia in CCRL2 KO mice develop a profound M1 phenotype compared to wild type (WT) mice after induction of EAE (38). These results highlight a potential role of CCRL2 in EAE-associated inflammatory responses and as such, provide a new potential target to control neuroinflammation.

Finally, using a neuron/microglia co-culture system, Yang et al. found that CCL2/MCP-1 (40) was able to activate microglia and stimulated production of pro-inflammatory cytokines such as TNF-α and IL-1β (41).

### Fibrosis

Upon infection, activated macrophages use a set of innate immune defense strategies such as phagocytosis, release of proteases and production of antimicrobial mediators, such as reactive oxygen and nitrogen species. An important side effect of this efficient inflammatory response is partial tissue destruction, which is normally followed by a repair response to regenerate the tissue (42). However, when this repair phase is persistent, it leads to fibrosis or so-called scarring of the tissue, which is defined by the accumulation of excess extracellular matrix components. In the end, this causes progressive loss of function of the affected organ(s) (43, 44). Alternatively activated (M2) macrophages are known to play an important role in wound healing and acquire a pro-fibrotic phenotype (45, 46). Since this phenotype is observed during the peak of the fibrotic immune response, it is suggested that such M2 macrophages are important inducers and regulators of fibrosis (44). For instance, by producing transforming growth factor-β1 (TGF-β1), M2 macrophages directly stimulate collagen production in myofibroblasts (47, 48) and enhance the expression of tissue inhibitors of metalloproteinases (TIMPs) that block the degradation of extracellular matrix (ECM) (48). Additionally, M2-derived chemokines play a role in fibrosis. For instance, CCL18/PARC is pro-fibrotic by promoting collagen production in lung fibroblasts (49–51). Increased collagen deposition, in turn, can enhance CCL18 production in alveolar macrophages, thereby suggesting a positive feedback loop between alveolar macrophages and fibroblasts (50). In idiopathic pulmonary fibrosis (IPF), one of the most common types of interstitial lung disease, CCL18 levels correlated with severity of fibrosis (52). More recently, CCL18 was identified as a marker for early identification of progressive interstitial lung disease in systemic sclerosis (SS) (53). Pechkovsky et al. showed that the Th2 cytokines IL-4 and IL-10 induce M2 polarization of alveolar macrophages (54). Interestingly, IL-10 enhanced the IL-4-induced CCL18 expression (54).

Besides CCL18, also CCL2 directly mediates a pro-fibrotic effect on fibroblasts by affecting TGF-β signaling, which in turn stimulates collagen production (55). Mice lacking CCR2, the cognate receptor for CCL2, showed reduced infiltration of inflammatory macrophages in two models of hepatic fibrosis (56, 57). These CCR2^−/−^ mice also developed less severe pulmonary fibrosis (58). Macrophages derived from CCR2 KO mice showed reduced production of matrix metalloproteinase (MMP)-2 and MMP-9 (59). Finally, the CCL2-CCR2 axis in macrophages has also been found to be important in renal fibrosis, where mononuclear cell infiltration and expression of chemokine receptors CCR1, CCR2, and CCR5 was enhanced in a spontaneous model of lupus nephritis (60).

Interestingly, in a commonly used model of bleomycin-induced lung fibrosis, CCR4^−/−^ mice showed a decreased inflammatory and fibrotic response compared to WT mice. Further analysis revealed that CCR4 KO alveolar and bone marrow-derived macrophages exhibited a more pronounced M2 activation state, as evidenced by increased expression of the typical M2 markers ARG1 and “found in inflammatory zone 1” (FIZZ1). Further experiments showed that the CCR4 ligand CCL17/TARC (61) plays a role in CCR4-dependent M1 activation leading to iNOS induction and oxidative injury, thereby affecting the development of bleomycin-induced pulmonary fibrosis (62). Additionally, FIZZ1 activates fibroblasts and induces myofibroblast differentiation in bleomycin-induced pulmonary fibrosis (63, 64). Chvatchko et al. reported that CCR4^−/−^ mice were more resistant to the effects of LPS compared to CCR4 WT mice (65). Further analysis revealed that peritoneal macrophages from CCR4 deficient mice possess an altered phenotype, more resembling M2 macrophages with elevated secretion of type 2 cytokines/chemokines and FIZZ1 protein (66). This study underscores the possible role of CCR4 in M1 activation.

In two different murine models of liver fibrosis, Heymann et al. demonstrated a protective role for the CCR8 receptor. Interestingly, hepatic macrophages from CCR8 KO mice showed an altered phenotype with more pronounced dendritic cell-like characteristics and enhanced CCL3 secretion (67).

### Macrophage polarization by chemokines in metabolic disorders

Nowadays it is generally accepted that the immune system and metabolism are tightly connected and recent studies have demonstrated that macrophages, in particular, are critical effector cells in metabolic inflammation (68). Resident macrophages in the adipose tissue of lean mice constitute ~10–15% of the total cell population. These adipose tissue macrophages (ATMs) express predominantly M2 characteristics and were shown to be critical for maintaining insulin sensitivity in adipocytes (69, 70). Conversely, in obesity, a state of low-grade systemic inflammation (71), adipocytes secrete pro-inflammatory mediators, which recruit monocytes into the adipose tissue mainly via the CCL2-CCR2 and CCL5-CCR5 axis (72–74). During obesity the number of macrophages in white adipose tissue increases fourfold (69) and macrophages acquire an M1 phenotype that contributes to the pro-inflammatory environment (75). Via secretion of pro-inflammatory cytokines, M1 ATMs contribute to insulin resistance by counteracting the insulin sensitizing action of the adipokines adiponectin and leptin (69, 76, 77). More recently, it has been shown that macrophage polarization in obesity can also be modulated by chemokines and their receptors. Kitade et al. demonstrated that inactivation of CCR5 not only resulted in a reduced number of ATMs, but the recruited ATMs switched toward an M2 phenotype (73). Additionally, obesity-induced insulin resistance was attenuated in obese CCR5^−/−^ mice (73). The question how CCR5 regulates M2 polarization is still unanswered. Obese mice with a genetic deficiency in CCR2 showed a reduced number of ATMs combined with a decreased expression of pro-inflammatory genes, compared to matched WT mice (72). Besides the CCR2 and CCR5 ligands, a recent study showed that during obesity CXCL12 recruits macrophages via CXCR4 to the adipose tissue (78). Moreover, CXCL12-CXCR4 signaling induced M1 macrophage accumulation and blocking this signaling diminished secretion of pro-inflammatory cytokines and improved insulin resistance (79).

The recruitment of macrophages, which stimulate the development of insulin resistance in obesity, is also critical in associated metabolic comorbidities such as nonalcoholic fatty liver disease (NAFLD) and nonalcoholic steatohepatitis (NASH). NAFLD is characterized by excessive fat accumulation in the form of intrahepatic triglycerides in the liver. NAFLD exhibits as a spectrum ranging from steatosis of the liver to a more necro-inflammatory form, NASH, which may develop into hepatic fibrosis, cirrhosis, or hepatic carcinoma (80). In the liver, macrophages consist of distinct populations, namely the resident, self-renewing Kuppfer cells and the inflammatory monocyte-derived macrophages (81–83). Kuppfer cells line the liver sinusoids and are involved in cholesterol metabolism by taking up and clearing modified low-density lipoprotein (LDL) and bacterial endotoxins through their scavenger receptors (84).

In line with the improved insulin resistance in CCR2^−/−^ obese mice, also hepatic steatosis was ameliorated (72). Besides CCR2, Karlmark et al. found that the CX_3_CL1-CX_3_CR1 axis is involved in the differentiation and survival of intrahepatic monocytes (85). The CX_3_CR1-mediated survival depends on the activation of the anti-apoptotic protein BCL2. Furthermore, in the absence of CX_3_CR1, hepatic macrophages showed a more pro-inflammatory phenotype characterized by increased TNF-α and iNOS production. These *in vivo* findings confirm earlier published data on elevated *Tnf*α expression and reduced *ARG1* expression in CX_3_CR1-deficient macrophages in a carbon tetrachloride (CCl_4_)-induced NAFLD mouse model (86). The increased pro-inflammatory response of liver macrophages was associated with enhanced liver fibrosis (85). This latter observation suggests that activation of the CX_3_CL1-CX_3_CR1 axis can work as an antifibrotic liver therapy.

### Macrophage polarization in cardio-vascular diseases

Cardiovascular disease (CVD) is the most common cause of mortality worldwide and accounts for 45% of all deaths in Europe (87). Atherosclerosis, an arterial narrowing due to plaque formation, is most often the underlying cause of myocardial infarction (88). The starting point of this pathology is the accumulation of lipoprotein particles in the intimal layer of the blood vessel. These lesions are mostly found at arterial branching points and bends, which are especially prone for local endothelial cell dysfunction. The stored lipoproteins are modified by several mechanisms such as oxidation, enzymatic processing, desialylation and aggregation, become pro-inflammatory and activate surrounding endothelial cells. Activated endothelial cells, in turn, release chemokines which recruit monocytes into the intimal and subintimal space of the artery where they differentiate into macrophages (89). These macrophages actively ingest cholesteryl ester-rich lipoproteins and eventually become “foam cells.” Although the uptake of lipoproteins by macrophages seems to be beneficial, these “foam cells” aggravate the disease through their secretion of pro-inflammatory mediators including cytokines and ROS and finally through their eventual death by necrosis or apoptosis. These latter processes result in the release of lipids and the formation of a pro-thrombotic core, which is a key-component of unstable plaques. Rupture of these plaques leads to the initiation of thrombosis, which limits or even blocks the flow of oxygen-rich blood to organs and other parts of the body (90, 91).

The first chemokine implicated in atherosclerosis was CCL2, which is normally not found in the blood vessel wall, but is induced in the early phase of atherosclerosis (92–94). Evidence for a prominent role of the CCL2-CCR2 axis came from a study by Boring et al. who reported that CCR2^−/−^ mice exhibit severely reduced atherosclerotic lesions (95). Later on, CXCR2, CX_3_CR1 and CCR1 have been implicated in monocyte/macrophage accumulation in atherosclerotic plaques (96, 97).

Relatively large numbers of pro-inflammatory macrophages were found in plaques and M1 macrophages are associated with unstable plaques (98, 99). M2 macrophages have only been detected later on and are more common in asymptomatic lesions and the stable zones of plaques (100). In addition to M1 and M2 macrophages, atherosclerotic plaques also contain specific macrophage subtypes, which are different from the phenotypes suggested by the classical activation model. For instance, in mice, oxidized lipids induce a distinct proatherogenic phenotype, referred to as Mox macrophages (101). These are characterized by reduced phagocytic and chemotactic capacities compared to M1 and M2 macrophages (101). So far, this phenotype is only observed in mice, whether Mox macrophages are also present in human lesions remains to be investigated. Upon intraplaque hemorrhage, due to rupture of invaded microvessels in the plaque, red blood cells lyse quickly and release hemoglobin and free heme. These heme products can directly polarize macrophages toward the Mhem or M(Hb) phenotype. Functionally, these subtypes are resistant to lipid accumulation and foam cell formation (102). Macrophage polarization to the M(Hb) phenotype occurs via exposure to the hemoglobin-haptoglobin complex (102, 103). This M(Hb) subset expresses high levels of the scavenger receptors CD163 (the hemoglobin-haptoglobin complex receptor) and CD206 (the mannose receptor) and is resistant to cholesterol accumulation because of the increased expression of the cholesterol efflux receptors ABCA1 and ABCG1 (104). Heme induces atheroprotective Mhem macrophages, which have high levels of HMOX1 (105) and are able to engulf extravasated erythrocytes (erythrophagocytosis) (106).

Besides lipids and their derivatives, heme products and also chemokines and growth factors present in atherosclerotic lesions can contribute to macrophage phenotype determination. During the early atherogenic phase, platelets can adhere and act as a rich source of chemokines. The platelet-derived chemokine CXCL4/PF-4 (107), similar to M-CSF, has been shown to prevent monocyte apoptosis and to promote the differentiation into macrophages *in vitro* (8). Later on it was found that CXCL4-induced macrophages acquire a specific phenotype, with a mixture of M1 and M2 characteristics and distinct from their M-CSF-induced counterparts. These so-called M4 macrophages express the pro-inflammatory chemokines TNF-α and IL-6, MMP-7, and MMP-12 and the calcium binding protein S100A8 (108, 109). The complete loss of the hemoglobin-haptoglobin scavenger receptor CD163, which is required for effective hemoglobin clearance after plaque hemorrhage (108, 110) and low expression of the antigen-presenting molecule HLA-DR (8) are typical characteristics of these so-called M4 macrophages. When hemoglobin or the hemoglobin-haptoglobin complexes bind the CD163 receptor, the atheroprotective HMOX1 is induced. Consequently, HMOX1 activity is also completely abolished in CXCL4-stimulated monocytes (111). Interestingly, the marked downregulation of CD163 and the novel phenotype induced by CXCL4 was reported to be irreversible (108). The presence of M4 macrophages within human atherosclerotic lesions is associated with advanced plaque morphology (112). M4 macrophages can be considered pro-atherogenic, since these may promote destabilization of the plaque fibrous cap (113).

**Figure 2:**
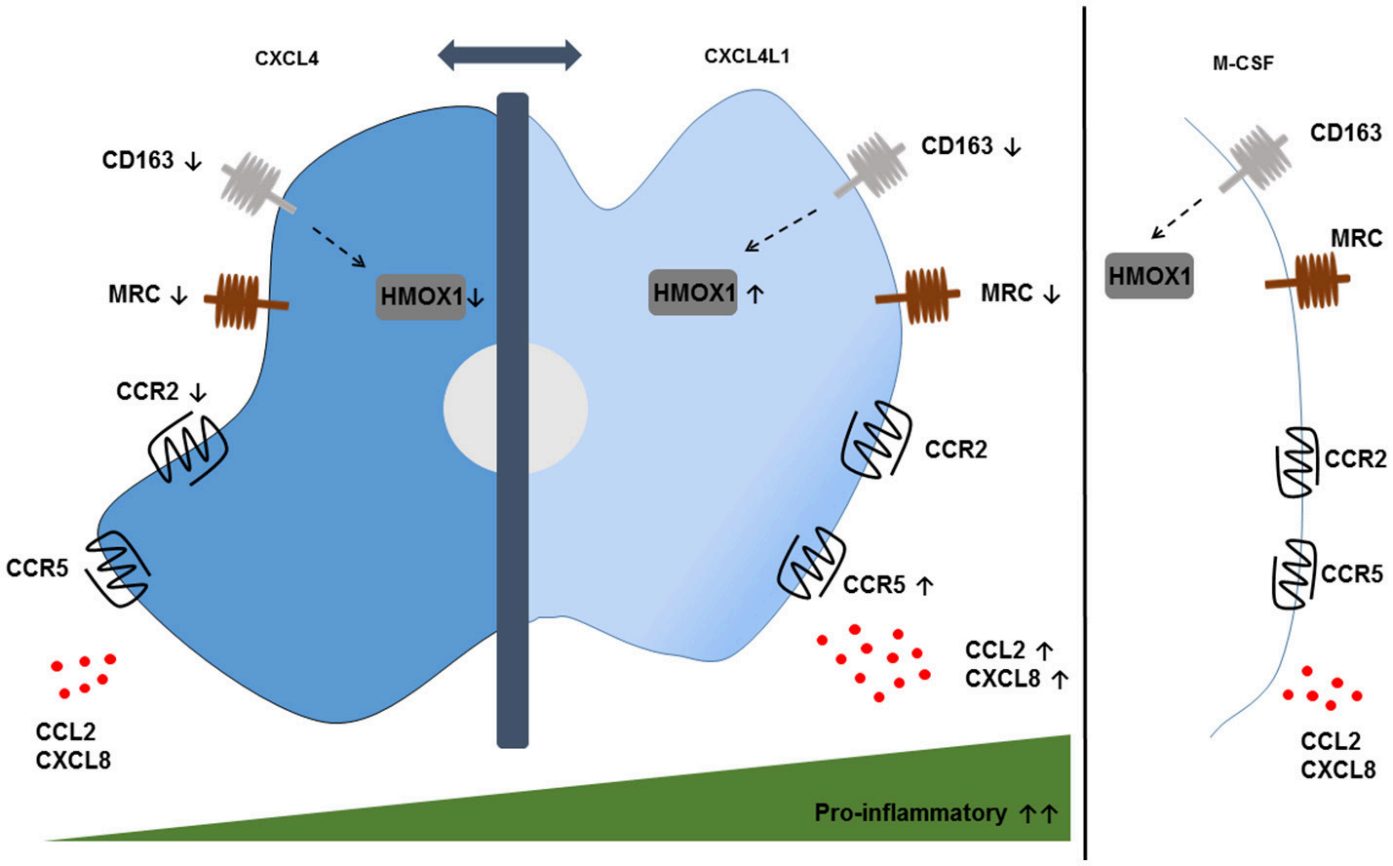
Phenotypic features of CXCL4- and CXCL4L1-induced macrophages. CXCL4-induced macrophages display a pro-atherogenic phenotype, characterized by the downregulation of the hemoglobin-haptoglobin scavenger receptor CD163 and the consequent downregulation of the HMOX1 enzyme compared to M-CSF-treated monocytes. Remarkably, the downregulation of HMOX1 is not observed in CXCL4L1-induced macrophages, which also show reduced expression of CD163. Both phenotypes show a downregulation of the mannose receptor (MRC) CD206. The expression of the chemokine receptors CCR2 and CCR5 and the secretion of pro-inflammatory chemokines CXCL8 and CCL2 are higher on CXCL4L1-treated monocytes compared to CXCL4-stimulated monocytes, thereby indicating more pro-inflammatory characteristics for CXCL4L1- than CXCL4-stimulated monocytes.

More recently, our group studied the effect of CXCL4L1/PF-4var (114), the non-allelic variant of CXCL4, on the differentiation of monocytes into macrophages (Figure 2) (115). Both variants are secreted by activated platelets and differ only in 3 amino acids near the carboxy-terminal end. The unique 3D structure of CXCL4L1 results in a decreased affinity for glycosaminoglycans (GAGs) and a more outspoken angiostatic potential compared to CXCL4 (116). Differently to M-CSF and CXCL4, CXCL4L1 is not a survival factor for monocytes. CXCL4L1-exposed monocytes display higher expression levels of the inflammatory chemokine receptors CCR2 and CCR5, suggesting that CXCL4L1 promotes a higher responsiveness to inflammatory chemokines, such as CCL2 and CCL3. Additionally, significantly higher amounts of CCL2 and CXCL8 (M1 marker) were measured in CXCL4L1-stimulated monocytes, whereas CXCL4 did modulate chemokine production in the same way as M-CSF. Finally, we found a lower expression of *IL-1 receptor antagonist (*IL-1RA) in CXCL4L1-treated monocytes, compared to CXCL4-treated monocytes, which is in line with the more inflammatory phenotype of macrophages generated in the presence of CXCL4L1 (115). Similar to CXCL4-treated monocytes, CXCL4L1-stimulated monocytes have a significantly lower expression of the CD163 receptor and the mannose receptor (MRC/CD206) compared to M-CSF treated monocytes (117). Interestingly, in contrast to M4 macrophages we found that *HMOX1* expression was significantly increased in CXCL4L1-treated monocytes (Figure 2) (115). So far, the role of CXCL4L1 in atherosclerosis is not further investigated. However, we showed that patients with stable coronary artery disease have a worse prognosis when CXCL4L1 levels in the serum are low (118).

### Role of TAMs in cancer

It is generally accepted that macrophages are the most abundant component of the leukocyte infiltrate that is influencing tumor development. Macrophages that infiltrate the tumor microenvironment are usually referred to as tumor-associated macrophages (TAMs) (119). TAM infiltration is correlated with a poor prognosis in numerous cancers, suggesting that they promote tumor progression (1, 81, 120, 121). Indeed, TAMs can stimulate proliferation, invasion, metastasis of tumor cells, promote angiogenesis and suppress the anti-tumor response (122). Poor anti-tumoral activities are a consequence of the higher production of IL-10, TGF-β and prostaglandin E2 (PGE2) and reduced synthesis of inflammatory cytokines such as TNF-α and IL-6. Furthermore, TAMs display poor antigen-presenting capacities, leading to suppression rather than stimulation of T cell activation and proliferation (13). The decreased production of inflammatory mediators in TAMs is associated with a defective nuclear factor-kappa B (NF-κB) activation in response to LPS and proinflammatory cytokines (123). In addition to the production of the most potent angiogenic factor VEGF, TAMs were shown to produce platelet-derived growth factor (PDGF) (13) and VEGF-C (124), which was suggested to play a role in peri-tumoral lymphangiogenesis and subsequent lymphatic metastasis. As such, TAMs are generally characterized as M2-like macrophages (125).

However, extensive TAM density is associated with increased survival in some specific tumor types. These findings suggest that TAMs comprise multiple distinct pro- and anti-tumoral subpopulations with overlapping features depending on different micro-environmental stimuli. In an explant model of colorectal cancer liver metastasis, CCR5 blockade with Maraviroc, a highly specific CCR5 inhibitor originally developed to treat HIV patients (126), induced a repolarization from an M2 toward an anti-tumoral M1-like phenotype (127). This phenotypic switch was mediated via increased levels of the signal transducer and activator of transcription 3 (STAT3), which is commonly linked to an M1 activation state, due to abrogation of the suppressor of cytokine signaling 3 (SOCS3) activity (128). This so-called re-education of macrophages induced by CCR5 inhibition in human cancer patients could possibly contribute to the further development of chemokine-based anti-cancer therapy.

**Figure 3:**
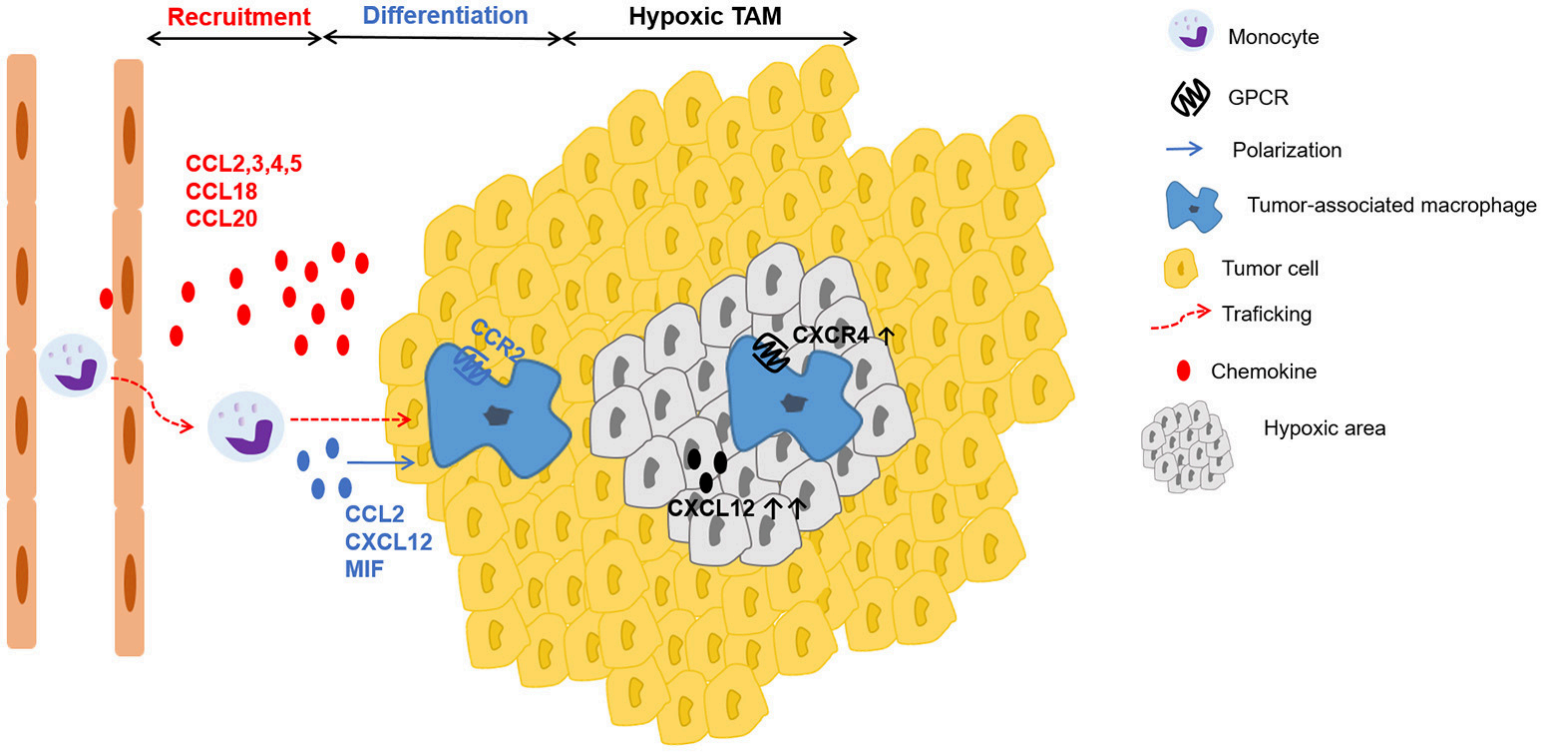
Schematic representation of chemokines involved in recruitment, differentiation and positioning of TAMs. Tumor-derived factors such as the chemokines CCL2, CCL3, CCL4, CCL5, CCL18, CCL20 actively recruit (red arrow) monocytes to the tumor, where they differentiate into tumor-associated macrophages (TAMs). In addition to several growth factors, a particular role in TAM polarization (blue arrow) has been described for the chemokines CCL2, CXCL12 and the chemokine-like protein MIF. In hypoxic areas, higher amounts of CXCL12 and increased expression of CXCR4 on macrophages enhance migration to and retention in these particular sites with low oxygen tension.

TAMs originate from circulating monocytes, which are recruited to the tumor by several growth factors and especially by chemokines, produced by stromal and tumor cells (120). Besides M-CSF, the CC chemokines CCL2, CCL3, CCL4, and CCL5 are well-recognized chemotactic factors for macrophage populations in the tumor (Figure 3) (129–133). CCL2 is dominantly expressed by many human carcinomas (134, 135) and detection of CCL2 in TAMs themselves even indicates the existence of an amplification loop for their recruitment (13, 136). Interestingly, once macrophages have entered the tumor microenvironment, the corresponding CCR2 is downregulated. It is suggested that receptor downregulation is a mechanism to trap recruited macrophages in the tumor micro-environment (137). Furthermore, in colon cancer models CCL20/LARC (138) chemoattracts monocytes that differentiate into TAMs. Additionally, in human breast cancer models CCL18 in collaboration with CSF-2 was involved in mobilization and recruitment of monocytes (139). Finally, VEGF-A was identified as a macrophage recruitment factor in an *in vivo* xenograft model, possibly acting indirectly through induction of chemoattractants (140).

Once differentiated, TAMs preferentially accumulate in the hypoxic areas of the tumor (141). Casazza et al. found that the protein Neuropilin-1 (Nrp-1) is essential for TAM mobilization toward Semaphorin 3A (SEMA3A), which is upregulated in hypoxic regions of the tumor. When TAMs enter these hypoxic areas, Nrp-1 expression is downregulated and TAMs are trapped in the hypoxic environment (142). Further, these hypoxic TAMs upregulate hypoxia-regulated genes and alter the gene expression profile, acquiring an even more pronounced pro-angiogenic, immunosuppressive, and pro-metastatic phenotype (143). This hypoxia-induced response is partly mediated via the key transcription factor hypoxia-inducible factor (HIF)-1α (144). Interestingly, in endothelial cells HIF-1α induces CXCL12 expression, which is in direct proportion to the oxygen tension in hypoxic areas (145). Additionally, hypoxia induces the expression of CXCR4 on monocytes and macrophages, thereby highlighting a possible role of the CXCL12-CXCR4 axis for TAM trafficking to the hypoxic tumor areas (Figure 3) (146).

Besides functioning as chemoattractants, some chemokines can also affect TAM polarization. Sierra-Filardi et al. disclosed an important role for the CCL2-CCR2 axis in regulating macrophage polarization, since blocking CCL2 led to an upregulation of M1 polarization-associated genes and decreased expression of M2-associated markers in human macrophages (147). Additionally, in several animal models of non–small-cell lung cancer (NSCLC) CCL2 blockade significantly reduced tumor growth. Although the total number of recruited macrophages did not change, there was a clear change in the polarization state of TAMs toward a more anti-tumor phenotype after CCL2 blockade (148). These results are in line with the findings from Roca et al. who showed that CCL2 stimulation shifts human peripheral blood CD11b^+^ cells toward a CD206^+^ M2-polarized phenotype (149).

Furthermore, in multiple myeloma (MM) CCL2, CCL3, and CCL14/HCC-1 (150) stimulate macrophage polarization into MM-associated macrophages (139), which induce MM drug resistance *in vitro* and in MM mouse models *in vivo* (151, 152). Tripathi et al. showed that hypoxic cancer cell-derived oncostatin M and the chemokine CCL11/eotaxin skewed macrophages toward an M2 phenotype (153, 154).

Besides factors produced by tumor cells, some chemokines produced by the macrophages themselves can affect their polarization. As such, autocrine CXCL12 production modulated the differentiation of monocytes toward a proangiogenic and immunosuppressive phenotype (155).

Interestingly, migration inhibitory factor (MIF), a cytokine that is not a chemokine but considered to be a “chemokine-like” molecule, was found to be a regulator of TAM polarization in melanoma bearing mice. A small molecule MIF antagonist attenuated tumor-induced macrophage M2 polarization coinciding with a reduced angiogenic potential (156).

The final step of cancer progression is metastasis, i.e., the dissemination of cancer cells from the primary tumor to distant organs. This highly complex process involves cell detachment from the primary tumor site, local invasion, intravasation into adjacent circulatory blood and lymphatic vessels, extravasation at distant capillary beds and proliferation in/colonization of distant organs (157). Before metastatic tumor cells are able to colonize, primary tumor-derived products prepare a primed microenvironment at secondary sites, also known as the pre-metastatic niche (158). Soluble factors including VEGF and placental growth factor (PIGF) induce the recruitment of VEGF-receptor 1 (VEGFR1) positive myeloid cells, which form clusters in the lungs and liver, preparing a permissive niche for disseminating tumor cells. Depletion of these VEGFR1^+^ cells inhibited metastasis (158). Disseminated cancer cells, in turn, produce CCL2 that recruits inflammatory CCR2^+^ monocytes from the blood to the metastatic niche, where they differentiate into so-called metastasis-associated macrophages (MAMs) (159). By secreting VEGF-A, these MAMs cause vessel wall permeabilization, allowing subsequent tumor cell extravasation (159). Interestingly, activation of CCR2 on MAMs induces the expression of CCL3 (160). CCL3 signaling via CCR1, in turn, promotes the retention of MAMs in the lung through vascular cell adhesion molecule (VCAM1)-α4 integrin mediated signaling and promotes cancer cell extravasation and retention at the metastatic site (160). Furthermore, VCAM 1 – α4 signaling protects cancer cells from pro-apoptotic signals (161).

Thus, TAMs and MAMs are not only a target for chemokines but also considered as a source of chemotactic mediators. Among these CCL2, CCL3, CCL17, CCL18, and CCL22 have been found to be produced by TAMs/MAMs (61, 162). In ascitic fluid from ovarian cancer patients CCL18, an attractant for Th2 cells was identified, but this chemokine was not produced by ovarian carcinoma cell lines *in vitro* (163). Therefore, it was suggested that the inflammatory mononuclear cells infiltrating the tumor were the CCL18-producing cells (164). Furthermore, CCL17 and CCL22 induce migration of regulatory T (T_reg_) cells via interaction with the CCR4 receptor (165). Thus, attraction of immunosuppressive immune cells through chemokine production is one of the pro-tumoral characteristics of TAMs.

## Concluding remarks

Monocyte-derived macrophages respond to a variety of stimuli to modulate their phenotype, which underlines their phenotypic plasticity, one of the major features of macrophages. M1 and M2 macrophages represent the extremities of a continuum of macrophage polarization states with M1 and M2 representing a rather pro-inflammatory and anti-inflammatory phenotype, respectively. Besides their well-known role in monocyte migration, chemokines have also been found to play a role in long-term regulatory processes by inducing macrophage differentiation and polarization in physiological and pathological processes.

## Author contributions

PR wrote the review and designed the figures; JVD and PP corrected the manuscript; SS provided critical feedback, helped shaping, and corrected the manuscript.

### Conflict of interest statement

The authors declare that the research was conducted in the absence of any commercial or financial relationships that could be construed as a potential conflict of interest.
